# “They just scraped off the calluses”: a mixed methods exploration of foot care access and provision for people with rheumatoid arthritis in south-western Sydney, Australia

**DOI:** 10.1186/1757-1146-6-34

**Published:** 2013-08-13

**Authors:** Gordon J Hendry, Kathryn A Gibson, Kevin Pile, Luke Taylor, Verona Du Toit, Joshua Burns, Keith Rome

**Affiliations:** 1 School of Science & Health, University of Western Sydney, Penrith, NSW, Australia; 2 Institute for Applied Health Research, Glasgow Caledonian University, Glasgow, G4 0BA, UK; 3 Department of Rheumatology, Liverpool Hospital, Sydney, NSW, Australia; 4 School of Medicine, University of Western Sydney, Campbelltown Hospital, Campbelltown, NSW, Australia; 5 Podiatry Department, Camden & Campbelltown Hospital, South Western Sydney Local Health District, Campbelltown, NSW, Australia; 6 School of Medicine, University of Western Sydney, Penrith, NSW, Australia; 7 The University of Sydney and Sydney Children’s Hospitals Network (Randwick and Westmead), Sydney, NSW, Australia; 8 Department of Podiatry, School of Rehabilitation & Occupation Studies, Faculty of Health & Environmental Sciences, Auckland University of Technology, Auckland, New Zealand

**Keywords:** Rheumatoid arthritis, Foot health, Podiatry, Footwear, Care access, Interpretative phenomenological approach, Qualitative, Cost of care

## Abstract

**Background:**

There is little indication that foot health services in Australia are meeting modern day recommendations for Rheumatoid Arthritis (RA) patients. The overall objective of this study was to explore the current state of foot health services for patients with RA with an emphasis on identifying barriers to the receipt of appropriate foot care in South-West Sydney, New South Wales, Australia.

**Methods:**

A mixed (quantitative and qualitative) approach was adopted. Indications for appropriate access to foot care were determined by comparing the foot health, disease and socio-demographic characteristics of patients with unmet foot care demands, foot care users and patients with no demands for foot care. Perceptions of provision of, and access to, foot care were explored by conducting telephone-based interviews using an interpretative phenomenology approach with thematic analysis.

**Results:**

Twenty-nine participants took part in the cross-sectional quantitative research study design, and 12 participants took part in the interpretative phenomenological approach (qualitative study). Foot care access appeared to be driven predominantly by the presence of rearfoot deformity, which was significantly worse amongst participants in the foot care user group (*p* = 0.02). Five main themes emerged from the qualitative data: 1) impact of disease-related foot symptoms, 2) footwear difficulties, 3) medical/rheumatology encounters, 4) foot and podiatry care access and experiences, and 5) financial hardship.

**Conclusions:**

Foot care provision does not appear to be driven by appropriate foot health characteristics such as foot pain or foot-related disability. There may be significant shortfalls in footwear and foot care access and provision in Greater Western Sydney. Several barriers to adequate foot care access and provision were identified and further efforts are required to improve access to and the quality of foot care for people who have RA. Integration of podiatry services within rheumatology centres could resolve unmet needs of people with RA by permitting rapid access to expert-led multidisciplinary foot care for people with RA.

## Background

In spite of the widespread recognition of the importance of foot care for patients with rheumatoid arthritis (RA), little is known about the availability and appropriateness of foot health services for RA patients in Australia. RA is the most common inflammatory arthritis with an estimated prevalence in Australia of 2–2.5% [1]. Foot problems including pain, joint stiffness and deformities are highly prevalent and affect the vast majority of patients with RA [2]. These problems are strongly associated with severe disability and impaired health-related quality of life (HRQoL) [3,4].

Research from the UK suggests that the provision of dedicated foot health services within rheumatology departments varies significantly by region [5]. Furthermore, reports suggest there is significant unmet demand for foot care amongst people with RA [5,6]. Similar findings have been reported in New Zealand, where 75% of people with RA and disabling foot problems had neither seen a podiatrist, nor received a foot assessment [7]. This suggests that discordance exists between health professionals’ perceptions of, and patients’ expectations for, appropriate foot care.

The minimally acceptable recommended standards of podiatric management for foot problems in RA include a detailed examination of the feet, therapy comprised of customised foot orthoses, exercise programmes, and advice concerning disease management, foot health and footwear [8,9]. Additionally, extended scope podiatry care may include corticosteroid injection therapies, musculoskeletal ultrasonography and instrumented gait analysis, where specialist training has been undertaken [8,9]. The aims of such interventions are to arrest inflammatory disease activity, relieve pain, maintain function, improve mobility, and prevent deformities in order to improve HRQoL [9].

Standards of care guidelines for people with inflammatory arthritis recommend that patients with early RA should be referred to podiatry for assessment, advice and intervention [10-12]. Moreover, expert-led recommendations advocate the integration of specialist podiatry within rheumatology multidisciplinary teams to allow rapid access to foot care [11,13-15]. There is evidence that such care paradigms are being implemented, through the support of academic-clinical partnerships and multi-centre research networks [8]. However, it is unclear whether foot care for RA patients in Australia is currently offered via integrated rheumatology-podiatry services, as has been recommended recently [16]. Lack of such integration suggests there may be a shortfall in foot care provision, as non-specialist podiatrists working in isolation may be unable to meet the complex needs of people who have RA.

Several barriers to accessing adequate allied health services have been described by patients with RA in Victoria, Australia including high costs of seeking care, and difficulties for patients of culturally and linguistically diverse backgrounds [17]. Indeed, it is probable that there are several barriers that may be preventing many people who have RA from accessing appropriate foot care across Australia. Accordingly, the aims of this study were to: 1) investigate whether or not foot care access is driven by foot health, disease and/or socio-demographic characteristics; 2) explore patients’ perceptions of foot health services for people who have RA in Greater Western Sydney; and 3) identify perceived barriers to adequate foot care access in this region.

## Methods

### Design

A mixed quantitative and qualitative methodological approach was used. Phase 1 of this study was a cross-sectional study design to address aim 1, while phase 2 was a qualitative study to address aims 2 and 3. The mixed methods approach was selected in order to clarify/elaborate upon the quantitative results of phase 1 through a more in-depth qualitative exploration [18].

### Phase 1

A cross-sectional design was adopted to compare the levels of foot health, disease, and socio-demographic characteristics in three groups (‘users’, ‘unmet demanders’ and ‘non-users’) of people with RA. The three groups were defined *a priori* based on the original work by Jacobi et al. [19]. A sampling frame was adopted to ensure similar numbers of participants were allocated to each group. Participants were assigned to the “users” if they received podiatry care in the previous 12 months and were satisfied with the number of visits; patients were assigned to “unmet demanders” if they did not receive podiatry care in the previous 12 months, and perceived an unmet demand for podiatry care; and patients were assigned to the group of “non-users” if they did not receive podiatry care in the previous 12 months and did not perceive an unmet demand for these services.

### Participants and setting

The research was conducted between May 2012 and February 2013, and the South Western Sydney Local Health District Research Ethics Committee granted ethical approval. Written informed consent was obtained from all participants. Participants were recruited consecutively from two South-Western Sydney outpatient rheumatology clinics based at Liverpool and Camden Hospitals respectively. Adult patients who met the American College of Rheumatology (ACR) Rheumatoid Arthritis Classification Criteria [20] were invited to participate.

### Measures

Demographic data including age, sex, marital status, education level and employment status were recorded. The primary outcome measure for phase 1 was the Foot Impact Scale for RA (FIS), a valid and reliable 51-item questionnaire with 2 subscales for impairment/footwear (FIS_IF_) and activity limitation/participation restriction (FIS_AP_) [21]. Foot pain was measured using a 100 mm visual analogue scale (VAS). Assessment of local disease activity was conducted by one of two experienced podiatrists (GJH/LT) through examination according to standard methods [22] of 18 joints of each foot (ankle, subtalar, calcaneo-cuboid, talonavicular, metatarsophalangeal, proximal interphalangeal, and distal inter-phalangeal joints). The foot joints were assessed for tenderness and/or swelling and recorded as present/absent before being summated to give a total score for both feet (0–36). Four foot and ankle tendons (tibialis posterior, flexor digitorum longus, flexor halluces longus, peroneus longus/brevis) and 3 miscellaneous soft tissues (Achilles tendon insertion, plantar fascia origin, and retrocalcaneal bursa) were assessed for tenderness and/or swelling. Clinical features were recorded as present/absent, and summated to provide a total score for both feet (0–14). Foot deformity score was recorded using the Structural Index (SI), a semi-quantitative scale for scoring rearfoot (0–14), forefoot (0–24) and combined foot deformities (0–36) in people who have RA [23].

Global functional impairment was measured using the Health Assessment Questionnaire (HAQ-DI), a valid, reliable and widely used instrument for measuring disability in adults [24]. The HAQ-DI includes an index of physical function ranging from 0 (best) to 3 (worst), as well as a 4 point Likert scale to measure ability to perform activities of daily living, a 100 mm VAS for global pain (0 represents no pain, 100 represents severe pain), and a 100 mm VAS for global health (0 represents very well, 100 represents very poor health).

The measure of socio-economic status was the Socio-Economic Index for Area (SEIFA) classification based upon postal area of residence [25]. This is a summary measure based upon data from the latest available Australian Census (2011). Subjects were allocated to one of 10 SEIFA categories, from the lowest decile (areas having the lowest incomes and highest proportion of unskilled workers) to the highest decile (areas having the highest incomes and highest proportion of professional/skilled workers).

### Phase 2

An interpretative phenomenological approach (IPA) was adopted to explore perceptions of foot health services and barriers to acceptable foot care access. An IPA is a philosophical approach which is based upon the exploration and understanding of lived experiences which may be used to answer questions that are important to a specific discipline [26]. The researchers’ experience, knowledge and standpoint are considered to be integral to the analysis and interpretation of the qualitative data through an IPA approach [26-28]. Semi-structured, telephone-based interviews were conducted to 1) permit respondents to be as comfortable as possible by remaining in their desired environment, and 2) to minimise the potential burden on participants by avoiding the need for attending further research appointments [29].

A sample size of 16 participants was targeted as is standard practice for the generation of qualitative data using an IPA [28,30,31]. Of the participants who were enrolled in phase 1, 29 were invited to participate in phase 2 and 16 agreed. Of those who agreed to participate in phase 2, 12 participants completed the study as two participants did not return their signed consent form, and two could not be contacted by telephone.

Provisional semi-structured interview scripts were developed by conducting a literature review [30]. Provisional scripts were reviewed and revised by all co-authors (5 podiatrists and 2 rheumatologists). Final topics for discussion included general experience of arthritis and foot problems, knowledge of available treatments and their effectiveness, opinions on foot care accessibility and acceptability, burden of disease and disease-related foot problems, and what improvements could be made to foot care services. Questions were open-ended to permit exploration and in-depth discussion [28,30,32] (see supplementary online material for interview script). All participants were contacted by telephone at their own home from a private office at the School of Science & Health, University of Western Sydney by a single researcher (GJH). Each interview was recorded via digital voice recorder (Olympus DS- 7000) and transcribed verbatim using a transcription kit (Olympus AS-7000).

### Statistical analysis

Analyses were performed using SPSS 19.0 for Windows (SPSS, Chicago, IL). Demographic characteristics were presented using descriptive statistics. In order to identify differences between the 3 patient groups for foot health, disease and socio-economic characteristics, Kruskal-Wallis with Mann–Whitney post-hoc testing were performed. The null hypothesis for the cross-sectional study (phase 1) was that there would be no significant difference between the three groups for foot health characteristics (measured using the FIS), indicating that foot health may not have an influence on foot care access and that access may be driven by less appropriate outcomes (socio-demographic/socio-economic characteristics). Results were considered statistically significant if *p*-values were less than 0.05.

### Qualitative data analysis

Thematic analysis of qualitative data was adopted, where two researchers (GJH and LT) searched for themes that emerged as important to the description of the phenomenon [33]. A data-driven inductive approach to coding and theme development was adopted, where a seemingly important item was coded prior to interpretation [34]. Final coding was reached by consensus between the two researchers (GJH and LT). Excerpts have been selected that represent the truthfulness of the data and the most expressive articulation of each theme [28,35]. Each participant was invited to read and verify the transcripts to support the trustworthiness of the data [30,35].

## Results

### Phase 1

**Figure 1:**
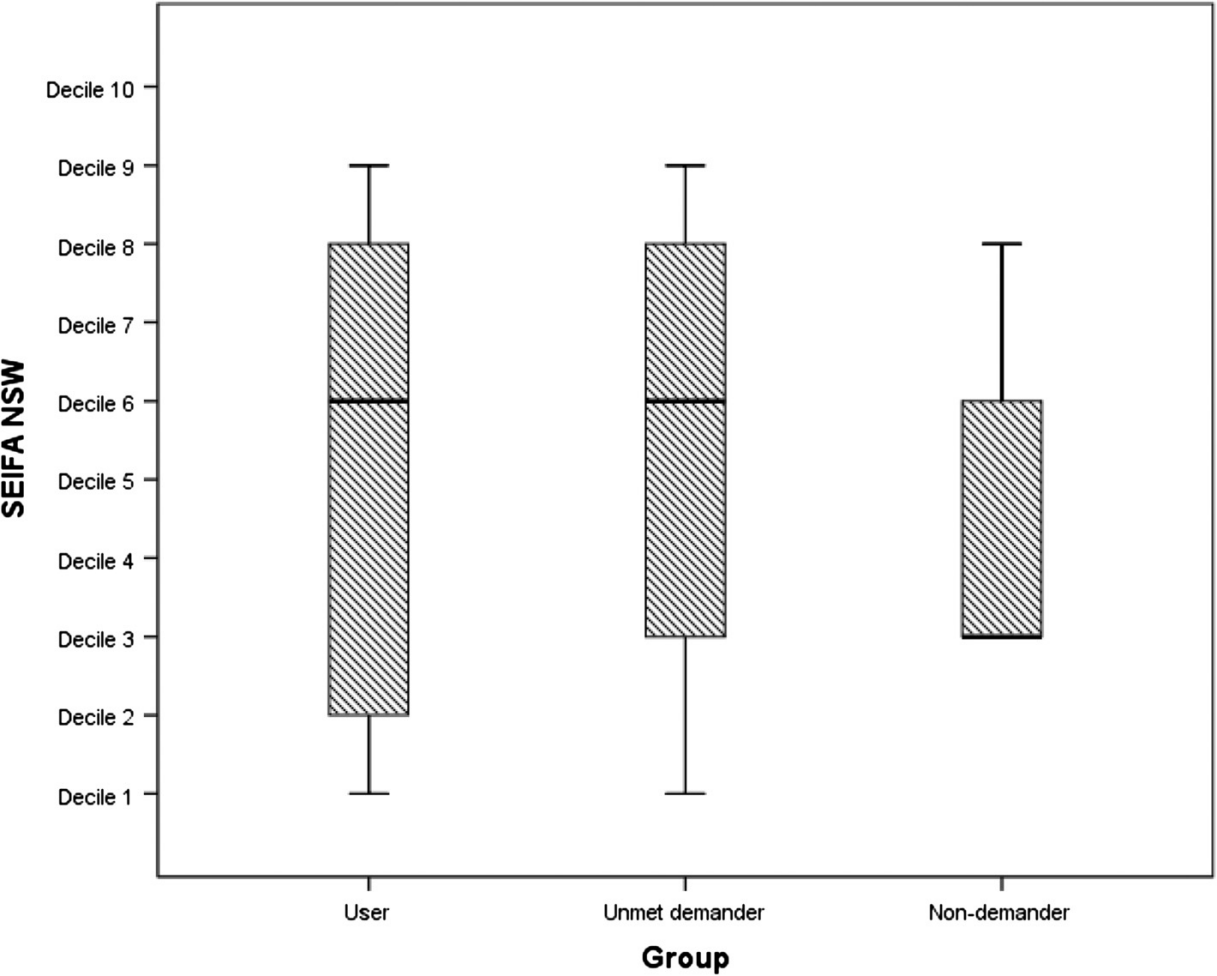
Box-and-whisker-plot of the median (central line), inter-quartile rage (horizontal box edge lines), and range (whiskers), for the Socio-Economic Index for Area classification for each study group (Decile 1, lowest socio-economic status to Decile 10, highest socioeconomic status.

Socio-demographic and socio-economic characteristics of phase 1 study participants are presented in Table 1. Groups were similar in terms of age, and marital status. Greater mean (SD) disease durations were observed in the user group participants (21.3 (12.2) versus 11.5 (6.7) and 9.7 (9.6) in the unmet demander and non-demander groups respectively]. No significant differences were observed between groups for education level and employment status (*p* > 0.05). Participants in the user- and unmet-demander groups were typically from areas rated in the 5th decile for socio-economic disadvantage (Figure 1). The non-demanders group were from areas rated in the 3rd decile for socio-economic disadvantage.

**Table 1:** Phase 1 (clinical survey component) characteristics of the study subjects, by study group

	**User**	**Unmet demander**	**Non-demander**	***P***
	**(n = 8)**	**(n = 10)**	**(n = 11)**	
	**Mean (SD)**	**Mean (SD)**	**Mean (SD)**	
Age, years	58.6 (10.1)	62.1 (12.6)	59.6 (9.6)	0.857
Sex, n				
Female	8	8	8	0.300
Male	0	2	3	-
Disease duration, years	21.3 (12.2)	11.5 (6.7)	9.7 (9.6)	0.087
Marital status, n				
Single	3	5	4	0.797
Married	5	5	7	-
Cohabiting	0	0	0	-
Education level, n				
Low	0	2	2	0.243
Medium	5	7	4	-
High	3	1	5	-
Employment status, n				
Full-time	1	2	3	0.539
Part-time	2	0	1	-
Unemployed	3	3	4	-
Retired	2	5	3	-
SEIFA Australia	5.5 (1.5–8)	4.5 (1.75–8)	3 (2–5)	0.746
SEIFA NSW	5.5 (1.5–8)	5.5 (2.5–8)	3 (3–6)	0.806

*SEIFA* Socio-Economic Index for Areas classification by postal area, *NSW* New South Wales, *IQR* inter-quartile range.

Disease characteristics of study participants are presented in Table 2. Non-significant differences were observed for all local and global disease characteristics (*p* > 0.05) except foot deformity. Statistically significant differences were observed between groups for rear- and combined fore- and rear-foot deformities using the SI (*p* = 0.012 and *p* = 0.019). Post hoc tests demonstrated that more severe rearfoot foot deformities were observed in the user group than the unmet demander group (*p* = 0.037), and more severe rearfoot and combined foot deformities were observed in the non-demander group (*p* = 0.005 and *p* = 0.004 respectively).

**Table 2:** Phase 1 (clinical survey component) disease characteristics of study subject, by group

	**User**	**Unmet demander**	**Non-demander**	***P***
	**(n = 8)**	**(n = 10)**	**(n = 11)**	
	**Mean (SD)**	**Mean (SD)**	**Mean (SD)**	
FIS IF (0–21)	13 (4)	10 (6)	13 (4)	0.354
FIS AP (0–30)	22 (4)	13 (11)	18 (8)	0.161
Foot pain VAS (0–100 mm)	40 (14)	29 (29)	36 (23)	0.488
HAQ (0–3)	1.5 (1.25)	1 (1)	1 (0.5)	0.218
HAQ ADL (0–4)	1.5 (1)	1 (1)	1.5 (1)	0.965
HAQ Pain VAS (0–100)	70 (30)	40 (32)	56 (23)	0.087
HAQ Health VAS (0–100)	51 (33)	35 (25)	56 (31)	0.153
Tender joints (0–36)	5 (4)	4 (5)	8 (11)	0.550
Swollen joints (0–36)	4 (3)	3 (2)	5 (5)	0.987
Tender soft tissues (0–14)	2 (2)	2 (2)	3 (3)	0.848
Swollen soft tissues (0–14)	1 (1)	0 (0)	1 (2)	0.096
SI Forefoot (0–24)	14 (7)	6 (6)	11 (7)	0.053
SI Rearfoot (0–14)	10 (3)†	5 (2)	5 (4) ‡	0.019
SI Total (0–36)	24 (9)	11 (6)	16 (8) ‡	0.012

*FIS* Foot Impact Scale, *IF* impairment/footwear, *AP* activity limitation/participation restriction, *VAS* visual analogue scale, *HAQ* Health Assessment Questionnaire, *ADL* activities of daily living, *SI* Structural Index.

† post-hoc Mann–Whitney test, *p* < 0.05 versus the unmet demander group.

‡ post-hoc Mann–Whitney test, *p* < 0.05 versus the user group.

### Phase 2

Demographic details of phase 2 study participants are outlined in Table 3. Mean (SD) interview duration was 38 (9) minutes. Twelve adult females (aged 44–83 years old) with a disease duration ranging from 3–34 years consented to participate in the study. Five reoccurring themes emerged from the data: 1) impact of disease-related foot symptoms; 2) footwear difficulties; 3) medical/rheumatology encounters; 4) foot and podiatry care access and experiences; and 5) financial hardship.

**Table 3:** Phase 2 (qualitative component) participant demographic data

**ID**	**Age (y)**	**Sex**	**Foot care status**	**Marital status**	**Disease duration (y)**	**Education status**	**Employment status**	**Rheumatology centre**
1	44	F	User	Married	3	Secondary	Unemployed	Liverpool
2	79	F	User	Married	30	College/Uni	Retired	Camden
3	53	F	User	Married	8	College/Uni	Part-time	Camden
4	55	F	User	Single	20	Secondary	Retired	Camden
5	55	F	User	Married	34	Secondary	Part-time	Liverpool
6	53	F	Unmet demander	Single	10	Secondary	Retired	Camden
7	58	F	Unmet demander	Single	5	Secondary	Unemployed	Liverpool
8	59	F	Unmet demander	Single	31	Secondary	Retired	Camden
9	58	F	Unmet demander	Single	8	Primary	Unemployed	Liverpool
10	65	F	Unmet demander	Married	24	Secondary	Retired	Camden
11	83	F	Unmet demander	Married	4	Secondary	Retired	Liverpool
12	59	F	User	Single	32	College/Uni	Part-time	Camden

*ID* unique participant identification number, *F* female, *y* years.

#### *Impact of disease-related foot symptoms*

Foot pain was the most influential symptom experienced by study participants and was described as the worst aspect of the disease by the majority. All respondents identified that their foot pain had limited their mobility and their ability to perform routine activities.

“The feet are also like a crushing burning pain as well like my feet feel as though they”re getting crushed sideways.” (participant 4).

“I sat on my bottom and used my heels to go upstairs because my toes on my feet were aching and throbbing.” (participant 6).

Feelings of frustration and embarrassment were described, and several participants felt that foot pain impacted upon their social lives because they did not want to be seen. Particularly bad periods of foot pain were attributed to periods of activity and employment involving prolonged standing/walking.

“I used to get frustrated with the things I couldn’t do because of the effect the arthritis had on me, particularly my hands and my feet.” (participant 2).

“I cannot stand in one place for a long time. I’m in pain I don’t know…I’m embarrassed to go somewhere out you know.” (participant 9).

“I came home from work in the evening I was in tears, my feet were just that sore.” (participant 5).

Participants expressed that they had tried to resist giving in to their foot pain. This appeared to be coupled with an eventual realisation through experience that there were limits to the amount of activity that they could perform. Participants described that they learned to ‘ration’ their activities to avoid foot pain.

“I tried to push myself back then but it got to a point where you know you just get too fatigued.” (participant 1).

“Even now I can only do one big thing in a day. I can only go to the shops very quickly and do that, and then that’s it…that’s my thing for the day.” (participant 7).

#### *Footwear difficulties*

The vast majority of participants reported problems with finding comfortable and aesthetically acceptable shoes because of foot problems such as foot deformity. Shoes made from softer materials were preferred as they provided better foot comfort. Open-toes shoes were preferred for a better fit, and also during the summer-time so that the feet did not overheat.

“I do have a lot of trouble with shoes, I have to have shoes that are very very soft.” (participant 3).

“In the summer I want my feet to breathe. It’s getting hot in the shoes, so I don’t wear them in the summer.” (participant 9).

“…the biggest problem I had getting shoes was the depth in the shoes, they weren’t tall enough around the toe area to accommodate the claw toes.” (participant 5).

Several respondents also described adaptation of their footwear purchasing habits in order to feel more comfortable. These adaptive strategies included purchasing shoes that were larger sizes compared to their usual sizes, females predominantly buying and wearing men’s shoes, and a preference for wearing slippers.

“…I’ve had to dress from the bottom- up…instead of going into a dress-shop first and then going for the shoes.” (participant 12).

“I’ve had to go up 1 shoe size and I’m like a double to triple E fitting in the shoes.” (participant 3).

“I just used to mainly wear men’s slippers.” (participant 2).

Respondents had purchased or received custom-made shoes and seemed to consider them to be useful in improving comfort. Two participants had received financial support in order to purchase their custom-made shoes, while others incurred the full cost. One participant was involved in the design of her shoes and expressed positive thoughts about a trade-off between comfort and her influences on their design.

“He put orthotics in them and the orthotics are a very soft material. And the shoes are very soft, so it’s much easier to walk with.” (participant 2).

“Between us we were able to create a shoe that looked decent. Didn’t look like an orthotic type shoe, it was appropriate for business wear um…hideously expensive but I knew that was going to be the case.” (participant 5).

“Thank goodness for the government, they made me a pair of shoes through the Enable [scheme]” (participant 9).

#### *Medical/rheumatology encounters*

A major factor related to medical encounters was a delayed diagnosis following the onset of symptoms. Participants described suffering from their arthritis problems for several months before obtaining a definitive diagnosis and treatment.

“So I went to see a rheumatologist and he wasn’t convinced that it was RA for about another 3–4 months. So I was about close to 9 months since they finally diagnosed what it was.” (participant 1).

“I got to go back to the doctors and it took me a few times, at the end I said I want a blood test to see where is come the pain from. And then eh….they said to me oh you’ve been diagnosed with arthritis.” (participant 9).

Participants expressed concern at the lack of interest in their feet by their doctors. They commented on an apparent lack of advice regarding foot-related complaints, a lack of referral to podiatry services, and a lack of communication with health professionals who may have been able to help with relief of symptoms.

“You know he didn’t really say go find yourself…you know…like a podiatrist or someone to look at your feet you know. They weren’t really interested in the feet, I don’t know why.” (participant 1).

Some participants commented positively on the effectiveness of systemic medications, whereas several participants explained that they could not tolerate many therapies that were advised due to side effects such as nausea.

“But em, the most effective was the methotrexate. Once I went on that I seemed to improve.” (participant 2).

“I cannot take this drug anymore, which was the methotrexate because it was making me so sick for 2 days every week.” (participant 7).

Communication and being allowed sufficient time and opportunity to ask doctors all necessary questions appeared to be an important issue to participants. Some respondents indicated that they were sufficiently able to have all their questions answered, whereas some felt that they did not have sufficient opportunity which led to negative perceptions of the consultation.

“He always gives me plenty of time in a consultation and asks me any questions outside of my pain or medication that’s worrying me.” (participant 3).

“So you can’t get all your questions. Sometimes I forget. Sometimes I’ve had things in my mind to ask and it’s just sort of so rushed that you’re out the door and then you remember something.” (participant 1).

#### *Foot and podiatry care access and experiences*

Many participants who had used podiatry services expressed dissatisfaction with care they had received and also commented on a perceived lack of RA foot management expertise amongst the podiatry work force. Respondents described a superficial focus by their podiatrist on basic skin and nail care, which did not seem to be relevant in the context of their painful joint symptoms. There appeared to be an unmet need for foot care amongst several participants as their primary symptoms were not being sufficiently addressed.

“…they just…just scraped off the calluses at the bottom of my feet.” (participant 8).

“I have found also that there’s not many that know too much about rheumatoid…so I’m cautious of who I go to.” (participant 12).

“If their attitude changed and was more sympathetic about the joints in the feet, yes I would [attend podiatry]. But if they simply had the blunt tunnel vision of podiatry about nail care then no, I wouldn’t [attend].” (participant 5).

Some respondents expressed positive comments regarding quality of the foot care/podiatry they had received. Participants described some symptom relief in terms of ‘feeling better’, but there was no mention of improvements in specific symptoms such as foot pain. Having feet checked by the podiatrist was considered to be of value to some respondents.

“…they were very good. I really enjoyed it, just having a look at my feet. I don’t know exactly what they did to them, just you know it was nice getting them done.” (participant 11).

“So they look after that for me, and it feels good when you go in…you know you just feel like your normal, but when you’re coming out you feel like “oh dear that feels good”.” (participant 2).

Some respondents were unsure what treatment options they required for their foot problems.

There appeared to be a lack of knowledge regarding the role of podiatry specifically for people with RA.

“I mean I knew the podiatrist but I didn’t know they’ve got anything to do with rheumatoid arthritis.” (participant 9).

#### *Financial hardship*

Several participants were unemployed or retired, seemingly as a result of their disease and as such they perceived that their arthritis had led to lost income/earning potential. Moreover, respondents who were still working commented on lost income as a direct result of seeking care.

“I actually have to take time off work because I am the last appointment of the day, which still doesn’t fit within my working hours. So I actually have to take time off and lose money to go to the doctors.” (participant 3).

“I’m only on a part-pension and em…yeah as I said sometimes you’ve gotta pay for stuff and you just don’t have the money for it.” (participant 10).

Many respondents were persevering with their disease-related foot problems because they couldn’t afford foot care. Some participants conducted trade-offs between items that they normally included in their budget, in order to pay for foot care.

“…that’s probably why I’ve dropped back on the food a bit because yeah um, it’s just finding that little bit extra to get the feet done, more regular.” (participant 4).

“I could look at the phone book and there are podiatrists but the first thing you have to do is pay a large fee.” (participant 6).

Most participants expressed dissatisfaction with benefits schemes such as the Pharmaceutical Benefits Scheme (PBS) (a scheme designed to permit partial reimbursement of costs for medications on the schedule list) and the Enhanced Primary Care (EPC) programme (a programme that permits patients with certain chronic health conditions to receive partial reimbursement of costs for up to five appointments with allied health professionals). Many participants found these systems to be confusing, and some were not sure of their eligibility for such schemes. Respondents discussed the PBS safety net, where they had to pay large sums of money for their medications before being eligible for support for their prescriptions. Some participants were unaware that they were eligible for rebates following podiatry care through the EPC. Whereas other participants were of the opinion that the annual rebates allowance was not sufficient for their needs.

“I’ve been told I should go to a podiatrist, and have my feet checked and everything, but I’ve not been told I can get back anything that I go to them for.” (participant 10).

“I’m always having my feet looked at. Because I go regularly I of course use up my quota all the time, very quickly. I think you’re only about 6 a year or something I think it is?” (participant 12).

## Discussion

Phase 1 demonstrated that access to foot care by people who have RA may not be triggered by appropriate determinants such as local foot impairments, pain, and/or disease activity. We observed poorer foot health characteristics in those who had accessed foot care. However, the only significant difference between the three groups was for rearfoot and combined foot deformity scores measure using the SI [23]. Those in receipt of foot care had significantly more severe levels of rearfoot deformity than those who had an unmet demand, and those who did not have a demand for foot care. Severe foot deformity may be more likely to trigger referral to podiatry services. However, previous research has demonstrated that foot deformity measured using the SI does not predict foot-related impairment or disability in people with RA [36,37]. Participants who were foot care users had longer disease durations despite being of a similar age, indicating an early disease onset. Longer disease durations are associated with the development of foot deformity [37-40], which may partly explain why these participants had accessed foot care. In addition, a study from the UK reported that age of disease onset is independently associated with an increased likelihood of seeing a podiatrist [41]. This is an important finding as functional loss occurs early and may be irreversible, whereas foot deformity largely represents a later-stage outcome where joint destruction and functional loss has already occurred [42].

Phase 2 of this study revealed that participants experienced significant disease burden as a result of painful foot problems, footwear difficulties, seeking appropriate care, and financial hardship. The results demonstrated that there was a significant unmet need for the treatment of foot pain. Previous research has demonstrated that longer disease duration is predictive of foot pain severity [43], and that foot pain is independently predictive of foot-related disability

[36]. The omission of foot joint examinations from the disease activity score (DAS) 28, which is typically performed by rheumatologists, may result in underestimation of foot disease activity and joint damage [44]. As such, a lack of detailed foot examinations by rheumatologists may influence decision making regarding referrals to podiatry.

Difficulties finding appropriate footwear were expressed by participants. Similar findings have been demonstrated in previous studies where participants perceived footwear difficulties to be a significant contributor to the overall impact of the disease [32,45-47]. Participants placed a significant emphasis on their seasonal footwear choices. This is perhaps unsurprising given that all respondents were female. Different perceptions of disease impact related to footwear according to gender have been discussed in previous qualitative studies [32,47]. Several participants were content with wearing sensible shoes except during summer time when their feet tended to overheat. Similar findings have been previously reported [32]. Personal influence on design of custom-made shoes was valued, which has been cited as an important factor in previous studies [32,47]. At present, it is unclear whether people with RA have sufficient access to customised footwear in Australia.

Participants reported varying levels of satisfaction concerning their systemic disease management in relation to their foot problems. Delayed diagnosis of RA and a lack of interest in foot symptoms by GPs and rheumatologists highlighted discrepancies between some patients’ and practitioners’ expectations regarding appropriate management of disease-related foot problems. Moreover, several participants commented on communication with their doctor/rheumatologist, with many describing negative experiences concerning the amount of time spent on being able to ask questions. Recent research has highlighted that these factors are associated with anxiety, anger, and a lack of confidence in their GPs [48].

Dissatisfaction with podiatry care was commonly reported. In particular, an overemphasis on callus debridement and nail cutting was frequently described. Previous clinical trials have demonstrated that scalpel debridement of callus provides minimal clinical benefit in people with or without RA [49,50]. As such, we would cautiously recommend that debridement of callus should not be conducted routinely in the management of people with RA, except in those who may be at risk from ulceration. Participants commented positively with regards to regular foot checks, which is in accordance with previous reports of the perceived benefits of reassurance [48]. However, there were concerns with some podiatrists’ lack of disease-specific expertise. We can postulate that there may be a shortfall in provision of appropriate foot care for people with RA by podiatrists in this region of Australia.

Several barriers to foot care were expressed by participants. Podiatry-naïve participants were not aware of any potential benefits of foot care. This is a commonly reported problem in podiatric rheumatology research, and often results in poor recruitment rates and subsequently under-powered randomised trials of podiatric interventions [51]. Recently published qualitative research has identified similar barriers to accessing/receiving appropriate foot care which suggests commonalities between participants of studies in Australia and the UK. Respondents participating in studies based in the UK have expressed disillusionment with the provision of foot health education and the scope of podiatry which resulted in confusion [30]. Whilst other UK-based respondents have reported dissatisfaction due to rheumatology health professionals not having taken their foot problems seriously [52]. As such, a greater emphasis on raising awareness of foot problems and foot care for people with RA may be required in NSW and Australia generally.

The major barrier to foot care access identified in this study was financial hardship. RA is known to have a substantial economic impact on patients [53]. In Australia, 64% of people with arthritis reported that their condition put a strain on their finances [54]. Participants were largely unemployed or retired, and as a result there appeared to be a significant financial burden associated with seeking care. Several participants expressed confusion regarding their entitlements to rebates through the Enhanced Primary Care (EPC) programme. Other participants felt that the EPC did not permit sufficiently frequent access to foot care, or dissatisfaction with the level of reimbursement. Indeed a recent survey suggests that many allied health professionals including podiatrists charge above the rebate rate [55]. Therefore, people from a lower socioeconomic status may experience difficulties seeking appropriate care as a result of financial constraints. Interestingly, the majority of participants in this study were generally from areas classed as the 3rd-5th lowest deciles for socio-economic status in NSW. Although further research is required, there may be a pressing need to lobby for a more realistic and equitable system for arthritis patients to have better access to foot care services in Australia.

There are limitations to the current study as it was conducted in South-Western Sydney, and may not reflect opinions of people with RA in Australia. The study was completed by a small convenience sample of consecutive patients attending rheumatology outpatient clinics and may have been be vulnerable to recruitment bias. We acknowledge that the results of phase 1 do not necessarily constitute the actual triggers which led to participants accessing foot care. The outcomes in phase 1, which were measured cross-sectionally in this study were indicative of outcomes at the time of measurement, and may not have been the initial reason for referral to podiatry.

Participants from phase 1 who were non-users of foot care services were generally uninterested in participating in the qualitative phase, which was comprised of predominantly foot care users or those with an unmet need for foot care. We adopted a telephone-based approach to interviewing participants in phase 2. This approach, while advantageous in terms of convenience may also result in bias due to exclusion of potential respondents who do not have a telephone [29]. However none of the participants who declined to take part stated that they were unable to participate due to the lack of a telephone. Lastly, these findings are derived from respondents involved in the delivery or receipt of foot care through largely private podiatry clinics and may not be directly relevant to other alternatively funded health care systems.

## Conclusions

Foot care provision does not appear to be driven by appropriate RA foot health characteristics such as foot pain or foot-related disability. Explorations of patient perceptions of foot care have highlighted that there may be significant shortfalls in footwear and foot care access in this region of NSW. Several barriers to adequate foot care access and provision have been identified including lack of awareness of podiatry services, lack of appropriate expertise amongst podiatrists, and financial hardship. Additional work is required to improve access to and the quality of foot care for people who have RA in this region. This study suggests that multi-centre Australia-wide audit of foot care access and provision for people with RA is required. Integration of podiatry services within rheumatology centres could resolve unmet needs of people with RA by permitting rapid access to expert-led multidisciplinary care for people with RA.

## Competing interests

The authors declare that they have no competing interests.

## Authors’ contributions

GJH conceived and executed the study protocol (with contributions from KAG, KP, VdT, JB and KR). All co-authors contributed to the design of semi-structured interview scripts. GJH and LT interpreted the findings with assistance from all co-authors. GJH and LT drafted the manuscript and the final version was read and approved by all co-authors.
